# Tumor Suppressor Function of miR-127-3p and miR-376a-3p in Osteosarcoma Cells

**DOI:** 10.3390/cancers11122019

**Published:** 2019-12-14

**Authors:** Joerg Fellenberg, Burkhard Lehner, Heiner Saehr, Astrid Schenker, Pierre Kunz

**Affiliations:** 1 Center for Orthopedics, Trauma Surgery and Paraplegiology, University of Heidelberg, 69118 Heidelberg, Germany; Burkhard.Lehner@med.uni-heidelberg.de (B.L.); heiner.saehr@gmail.com (H.S.); Astrid.Schenker@med.uni-heidelberg.de (A.S.); pkunz2@ix.urz.uni-heidelberg.de (P.K.); 2 Clinic for Shoulder and Elbow Surgery, Catholic Hospital Mainz, Rhineland-Pfalz, 55131 Mainz, Germany

**Keywords:** osteosarcoma, cancer therapy, microRNA, miR-127-3p, miR-376a-3p, chorioallanthoic membrane assay

## Abstract

Since the introduction of high-dose chemotherapy about 35 years ago, survival rates of osteosarcoma patients have not been significantly improved. New therapeutic strategies replacing or complementing conventional chemotherapy are therefore urgently required. MicroRNAs represent promising targets for such new therapies, as they are involved in the pathology of multiple types of cancer, and aberrant expression of several miRNAs has already been shown in osteosarcoma. In this study, we identified silencing of miR-127-3p and miR-376a-3p in osteosarcoma cell lines and tissues and investigated their role as potential tumor suppressors in vitro and in vivo. Transfection of osteosarcoma cells (*n* = 6) with miR-127-3p and miR-376a-3p mimics significantly inhibited proliferation and reduced the colony formation capacity of these cells. In contrast, we could not detect any influence of miRNA restoration on cell cycle and apoptosis induction. The effects of candidate miRNA restoration on tumor engraftment and growth in vivo were analyzed using a chicken chorioallantoic membrane (CAM) assay. Cells transfected with mir-127-3p and miR-376a-3p showed reduced tumor take rates and tumor volumes and a significant decrease of the cumulative tumor volumes to 41% and 54% compared to wildtype cells. The observed tumor suppressor function of both analyzed miRNAs indicates these miRNAs as potentially valuable targets for the development of new therapeutic strategies for the treatment of osteosarcoma.

## 1. Introduction

Osteosarcoma is the most common primary bone tumor, typically arising from the metaphysis region of the long bones in children and young adults [1,2]. It is characterized by an aggressive growth behavior and early metastasis to the lungs. The introduction of neoadjuvant and adjuvant chemotherapy significantly improved outcomes, with long term relapse free survival rates ranging from 55% to 75%. However, the remainder of patients will relapse, most often with pulmonary metastases, poor response to chemotherapy, and a significant decrease of survival rates ranging from 20% to 30%. Unfortunately, no significant improvement of osteosarcoma therapy has been achieved within the last three to four decades [3,4,5]. Even the latest and largest multinational therapy-optimizing study “Euramos-1”, evaluating a multidrug chemotherapy regimen, could not contribute to an improved survival of osteosarcoma patients [6]. Thus, new therapeutic strategies replacing or complementing conventional chemotherapy are urgently required.

Attractive target molecules for the development of such new therapeutic strategies are microRNAs (miRNAs) [7,8]. MiRNAs are small non-coding RNA molecules regulating gene expression by degradation of the corresponding mRNA or inhibition of protein synthesis. MiRNAs are involved in the regulation of many important cellular functions, including cell cycle regulation, proliferation, differentiation, and apoptosis. As a consequence, aberrant miRNA expression patterns play a crucial role in various diseases, including cancer [9,10,11], by targeting oncogenes and tumor suppressors that influence angiogenesis, cancer-stem-cell biology, and drug resistance [12]. In osteosarcoma, several miRNAs have already been demonstrated to crucially regulate proliferation, migration, invasion, and apoptosis [13,14,15,16]. In addition, miRNAs have been identified that act as tumor suppressors and might serve as prognostic markers for osteosarcoma [17,18]. Further, miRNAs have been shown to be involved in the development of chemoresistance [19,20] and to be suitable as biomarkers for the early diagnosis of osteosarcoma [21,22].

Endogenous miRNA levels can be modified by several approaches. Chemically modified oligonucleotides can either be used to block miRNA function or microRNA mimics can be used to increase intracellular miRNA levels [23,24]. Besides the use as diagnostic or prognostic markers, aberrantly expressed miRNAs might thus also be valuable therapeutic targets.

MiR-127 and miR-376a have frequently been reported to be silenced in several types of cancer, suggesting pivotal roles for the development and progression of cancer [25,26,27,28,29,30,31,32]. We hypothesized that these miRNAs might also be silenced in highly malignant osteosarcoma, thus representing valuable targets for the improvement of current osteosarcoma therapy. To verify this hypothesis, we analyzed the expression of miR-127-3p and miR-376a-3p in osteosarcoma cell lines and tissue and investigated the impact of miRNA restoration on the neoplastic phenotype of osteosarcoma cells in vitro and in vivo. We confirmed the supposed downregulation of miR-127-3p and miR-376a-3p and observed a significant influence of these candidate miRNAs on proliferation and colony formation in vitro as well as tumor engraftment and growth in vivo. These data indicate that miR-127-3p and miR-376a-3p act as tumor suppressors and represent promising targets for osteosarcoma treatment.

## 2. Results

### 2.1. Silencing of miR-127 and miR-376a in Osteosarcoma Cell Lines and Tissue

**Figure 1:**
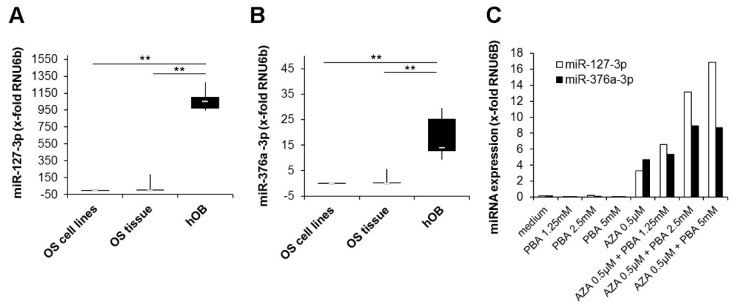
Silencing of miR-127-3p and miR-376a-3p in osteosarcoma cell lines and tissue. Quantitative real-time PCR analysis of (**A**) miR-127-3p and (**B**) miR-376a-3p expression in osteosarcoma cell lines (*n* = 7), osteosarcoma tissue (*n* = 8), and primary human osteoblasts (hOBs) (*n* = 5). Expression values were normalized to the expression of the small nuclear RNA U6 pseudogene (RNU6B). The white lines indicate the medians, the lower boundary of the box of the 25th percentile and the upper boundary of the box of the 75th percentile. The whiskers indicate the highest and lowest values. *p*-values were determined by the Mann–Whitney U test. (** *p* < 0.01). (**C**) Upregulation of miRNA expression by epigenetic modifiers. The osteosarcoma cell line 143B was treated for seven days with the indicated concentrations of 5′-Aza-2′-deoxycytidine (AZA) followed by a further three days of culture with or without the addition of phenylbutyric acid (PBA). After the incubation period, the expression of miR-127-3p and miR-376a-3p was quantified and normalized to the expression of RNU6B.

Expression of candidate miRNAs was analyzed by quantitative RT-PCR in seven cultured osteosarcoma cell lines and eight frozen osteosarcoma tissue samples. As controls, cultured primary human osteoblasts (hOBs) isolated from bone chips were used. Expression of both miRNAs was nearly undetectable in all osteosarcoma cell lines tested. In osteosarcoma tissues, the median miR-127-3p expression was 62-fold and the miR-376a expression 12-fold higher. We assume this increase to be due to non-neoplastic bystander cells within the tumor tissue. Compared to osteosarcoma cells and tissue, a considerably higher expression was observed in hOBs, with a 15000-fold higher expression of miR-127-3p and a 1700-fold higher expression of miR-376a-3p. (Figure 1A,B). To verify that epigenetic alterations account for the observed miRNA silencing, we treated 143B cells with the demethylating agent 5′-Aza-2′-deoxycytidine (AZA), the histone deacetylase inhibitor phenylbutyric acid (PBA), or a combination of both. Expression of both miRNAs could be induced by AZA and was further elevated by a combined treatment with AZA and PBA in a dose-dependent manner. Treatment with PBA alone showed no effect on miRNA expression (Figure 1C).

### 2.2. Restoration of miR-127-3p and miR-376a-3p Inhibits Proliferation of Osteosarcoma Cells

**Figure 2:**
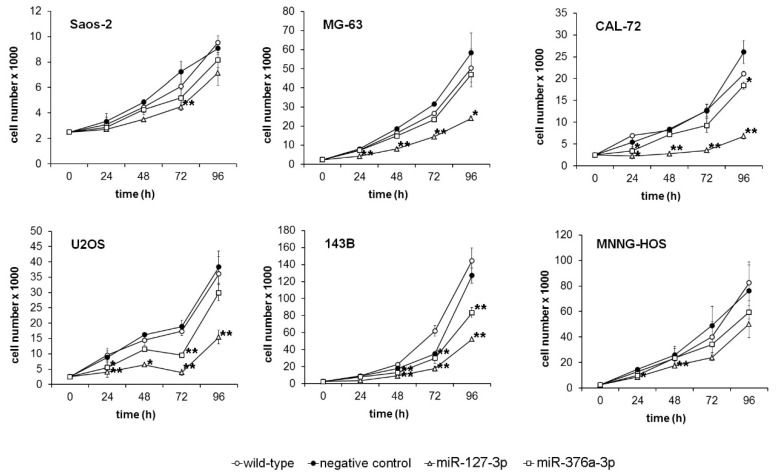
Restoration of endogenous levels of miR-127-3p and miR-376a-3p inhibits proliferation of osteosarcoma cells. Six osteosarcoma cell lines were transfected with miR-127-3p and miR-376a-3p mimics before cells were counted at the indicated time points. Non-transfected wildtype cells (WT) and cells transfected with a negative control miRNA (NC) served as controls. Experiments were done in triplicates. * *p* < 0.05, ** *p* < 0.01 compared to wildtype cells.

Six osteosarcoma cell lines were transiently transfected with miRNA mimics and transferred into 96-well culture plates 48 h after transfection. As controls, non-transfected wildtype cells (WT) or cells transfected with a negative control miRNA (NC) were used. Proliferation was analyzed by quantification of cell numbers after 0, 24, 48, 72, and 96 h. We observed a significant inhibition of the proliferation of miRNA-transfected cells that was most pronounced after restoration of miR-127-3p. While cell numbers decreased 96 h after miR-376a-3p restoration to 65% to 90% compared to cells transfected with the negative control miRNA, restoration of miR-127-3p induced a reduction to 26% to 79% compared to control cells. CAL-72 turned out to be the most sensitive cell line to miR-127-3p restoration, with an average reduction of cell numbers to 26%, followed by U2OS (40%), 143B (41%), and MG63 (41%). The cell line most sensitive to miR-376a-3p restoration was 143B (65%) followed by CAL-72 (71%) and MNNG HOS (79%). As a consequence, cell doubling times increased after miR-127-3p restoration 1.33-(Saos-2) to 3.1-fold (CAL-72), and after miR-376a restoration, 1.1- (MG63) to 1.74-fold (143B) compared to wildtype cells (Figure 2).

### 2.3. Mir-127-3p and miR-376a-3p Reduce the Colony Forming Capacity of Osteosarcoma Cells

**Figure 3:**
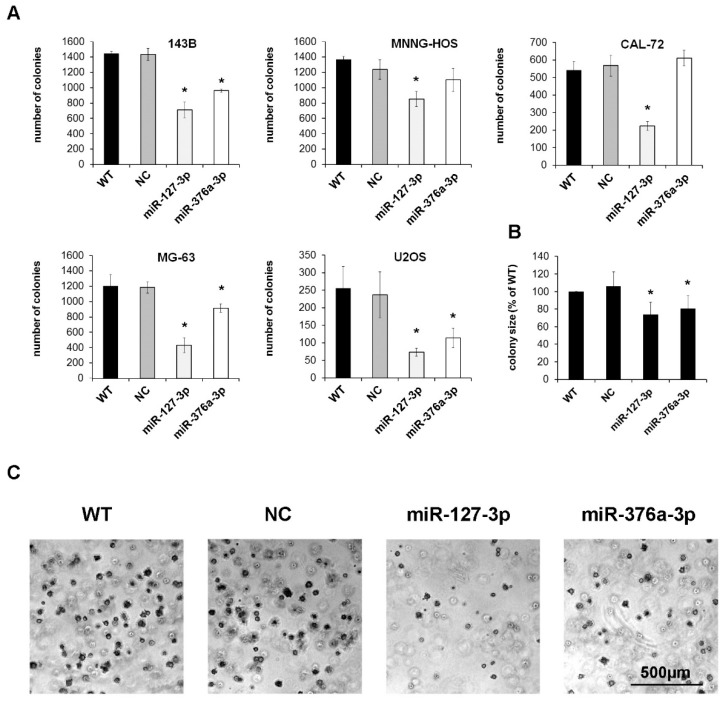
Mir-127-3p and miR-376a-3p reduce the colony forming capacity of osteosarcoma cells. (**A**) Cells transfected with miRNA mimics, a negative control miRNA (NC), or untreated wildtype cells (WT) were cultured for 10 days in soft agar, photographed, and colonies >500 µm^2^ were counted using ImageJ software (* *p* < 0.05 compared to WT group). (**B**) Mean colony size of all five osteosarcoma cell lines normalized to the wildtype cells. (**C**) Representative photographs of colonies formed by MG-63 cells.

The ability to form colonies in a semi-solid matrix is a key feature of neoplastic cells. We therefore analyzed the colony forming capacity of osteosarcoma cells in soft agar after transient transfection with miR-127-3p and miR-376a-3p mimics. Except Saos-2 cells, all cell lines tested formed high numbers of colonies as early as 10 days after plating. Restoration of miR-127-3p reduced the number of colonies to an average of 43% compared to untreated wildtype cells. Restoration of miR-376a was less effective, with an average reduction to 76%. (Figure 3A). Besides the colony number, both candidate miRNAs also significantly reduced the size of the colonies (Figure 3B,C).

### 2.4. Cell Cycle Analysis of Osteosarcoma Cells with Restored miRNA Expression

**Figure 4:**
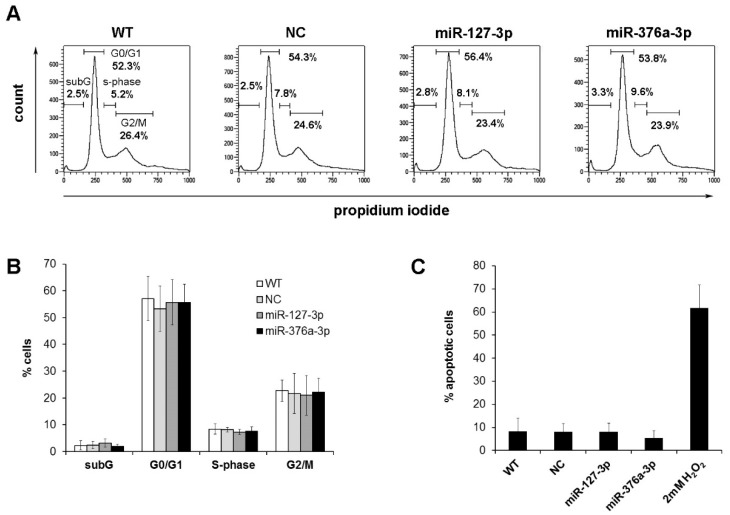
Cell cycle analysis of osteosarcoma cells with restored miRNA expression. The percentage of cells in the subG, G0/G1-, S-, and G2/M-phase was determined 96 h after transfection by flow cytometry. (**A**) Representative graphs of the cell line CAL-72. (**B**) Summary of the mean cell counts and standard deviations obtained from three independent experiments with six cell lines. (**C**) Percentage of apoptotic cells determined by NucView 488 staining 96 h after miRNA restoration. Positive control cells were treated with 2mM H_2_O_2_ for 4 h and further incubated for 24 h without the addition of H_2_O_2_.

The observed inhibition of proliferation and colony formation after miRNA restoration prompted us to analyze the influence of these candidate miRNAs on the cell cycle. We quantified cell numbers in the subG-, G0/G1-, S-, and G2/M-phase after transient transfection of candidate miRNAs. We could not detect any significant differences of cell numbers within the different cell phases after miRNA restoration (Figure 4A,B, Supplementary Figure S1). We further could not detect any significant differences within the subG fractions, indicating that miRNA restoration does not induce cell death. To verify this hypothesis, we analyzed caspase activation in cells transfected with the candidate miRNAs, showing no induction of apoptosis (Figure 4C).

### 2.5. Restoration of miR-127-3p and miR-376a-3p Inhibits Tumor Growth in Vivo

**Figure 5:**
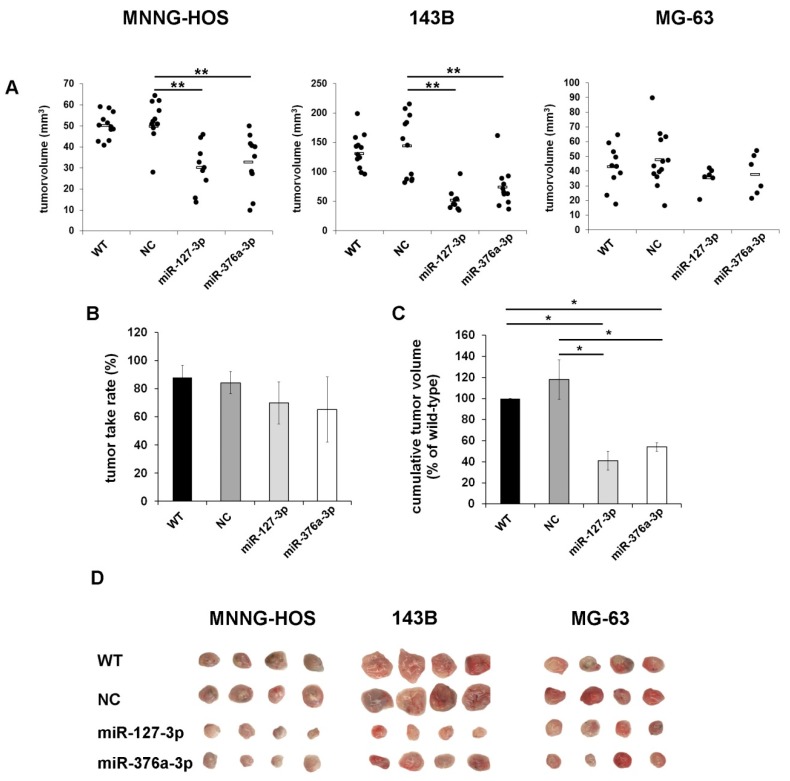
Restoration of miR-127-3p and miR-376a-3p inhibits tumor growth in vivo. Osteosarcoma cells were transiently transfected with miRNA mimics, a negative control miRNA (NC), or left untreated (WT). Then, 72 h after transfection, cells were transplanted onto the chorioallantoic membrane (CAM) of fertilized chicken eggs (*n* = 15 per group). Tumors were resected on day 16 and the tumor volumes (**A**), ** *p* < 0.01, the tumor take rates (**B**), and the cumulative tumor volumes (**C**) were calculated (* *p* < 0.05). (**D**) Representative photographs of tumor xenografts from each treatment group.

To analyze the effects of candidate miRNA restoration on tumor engraftment and growth in vivo, we used a chicken chorioallantoic membrane (CAM) assay that we recently optimized for the use with osteosarcoma cells [33]. Transiently transfected cells were immobilized within a basement membrane matrix derived from Engelbreth-Holm-Swarm mouse sarcoma (Cultrex BME Tye 3) that mimics the in vivo microenvironment and provides growth factors and nutrients for the first 72 h until tumor vascularization is established. The cell-matrix suspension was transplanted onto the CAM of fertilized chicken eggs on embryonic development day 9 (EDD 9) and incubated for one week until EDD 16. Formed solid tumors were resected, photographed, and tumor volumes and tumor take rates were calculated. All tested cell lines formed three-dimensional tumor xenografts, with volumes ranging from 15 to 200 mm^3^ depending on the cell line used (MNNG-HOS, 143B, and MG-63). However, both candidate miRNAs induced a significant reduction of tumor volumes (Figure 5A). While wildtype cells and cells transfected with a negative control miRNA formed tumors in 87.8% and 84.1% of all transplanted eggs, restoration of miR-127-3p reduced the tumor take rates to an average of 69.7% (MNNG HOS 64.7%, 143B 90%, MG63 54.5%) and miR-376a-3p reduced tumor take rates to an average of 65.2% (MNNG HOS 68.5%, 143B 91.7%, MG63 35.3%). Positive correlations of tumor take rates with the overall and event-free survival of patients have already been shown after transplantation of lung cancer tissue into mice. Metabolically more active tumors, tissues derived from metastases, as well as tumors with CD133+ cancer-initiating cells also showed higher frequencies of xenograft establishment, suggesting that the tumor take rate reflects the aggressiveness of the transplanted tumor [34]. Our observation of reduced tumor take rates after miRNA transfection thus indicates that miRNA restoration also influences the aggressiveness of the analyzed osteosarcoma cells.

The observed reduction of tumor take rates and tumor volumes resulted in a decrease of the cumulative tumor volumes from 887 and 990 mm^3^ in the wildtype and negative control group to 318 and 466 mm^3^ in the groups with miR-127-3p- and miR-376a-3p-transfected cells, corresponding to a reduction to 41% and 54% compared to wildtype cells (Figure 5B–D).

## 3. Discussion

Downregulation of miR-127 has been shown to be a common feature in several types of cancer, including ovarian cancer [27], gastric cancer [28], squamous cell carcinoma [26], pancreatic cancer [31], giant cell tumor of bone [35], and osteosarcoma [36]. Likewise, downregulation of miR-376a has been reported in multiple cancer types, including breast cancer [32], lymphoma [30], non-small-cell lung cancer [29], giant cell tumor of bone [35], and osteosarcoma [37]. In these studies, both miRNAs have been shown to inhibit proliferation, migration, and invasion of cancer cells and to induce apoptosis upon restoration.

Since miRNAs represent highly attractive targets for the development of new therapeutic strategies, and tumor suppressor functions of miR-127 and miR-376a have already been demonstrated in osteosarcoma cell lines in vitro [36,37,38], we aimed to validate these tumor suppressor functions in further osteosarcoma cell lines and further to analyze the impact of candidate miRNA restoration on tumor engraftment and growth in vivo.

Together with the mentioned reports from studies on other cancer types, our observation of miR-127-3p and miR-376a-3p downregulation in osteosarcoma cell lines and tissues strengthens the significance of miRNA silencing for the development and progression of cancer. Interestingly, miR-127-3p and miR-376a-3p are located in miRNA clusters within the imprinted Dlk1-Dio3 region on chromosome 14, which is regulated by DNA methylation and histone acetylation [39]. As a consequence, the expression of both miRNAs can be restored by the treatment of cancer cells with the DNA methyltransferase inhibitor 5-aza-2′-deoxycytidine or the histone deacetylase inhibitors phenylbutyrate (PBA) and trichostatin A, indicating that epigenetic alterations account for dysregulation of miR-127-3p and miR-376a-3p independent from the type of cancer [40,41].

Our observation of a significant inhibition of cell proliferation and colony formation after restoration of miRNA expression in vitro as well as the reduction of tumor engraftment and growth in vivo confirmed the tumor suppressor features of both candidate miRNAs in osteosarcoma that might thus represent valuable targets for novel therapeutic approaches. Interestingly, attenuation of proliferation was independent from cell cycle modifications and induction of apoptosis. Comparable observations have been made in pancreatic ß cells after elevation of miR-127 levels [42].

Several miR-127 and miR-376a target genes have already been identified. Therapeutic targeting of these candidate miRNAs would thus simultaneously influence multiple cancer-related pathways, potentiating the effectiveness of this therapy approach. Known miR-127 target genes include *ITGA6* (integrin alpha-6) [36], *SETD8* (lysine methyltransferase 8) [38], *WNT7A* (Wnt Family Member 7A) [28], *BAG5* (BCL2-associated athanogene 5) [43], and *COA1* (Cytochrome C Oxidase Assembly Factor 1 Homolog) [35]. *ITGA6* has been shown to enhance tumor invasion and the activity of tumor-initiating cells in metastatic breast cancer [44]. *SETD8* monomethylates lysine 20 of histone H4 and other proteins, including proliferating cell nuclear antigen (PCNA) and p53, that are involved in the regulation of several processes associated with tumor growth, including DNA replication, DNA damage response, transcription modulation, and cell cycle regulation [45]. Wingless-related integration site (Wnt) proteins are also known to play important roles in the regulation of cell proliferation, migration, and tumorigenesis through the Wnt/β-catenin signaling pathway. For example, small inhibitory RNA (siRNA)-mediated knock-down of *WNT7A* has been shown to significantly inhibit migration and invasion of gastric cancer cells [28]. *BAG5* has been reported to be overexpressed in several cancers, to inhibit stress-induced apoptosis [46], and to mediate its proto-oncogenic properties by stabilizing mutant p53 [47]. The miR-127 target gene *COA1* is located in the inner mitochondrial membrane and plays an important role in the assembly of Cox1 (cytochrome-c-oxigenase subunit 1) within the respiratory chain complex.

Likewise, the miR-376a target genes *MYC* [29], *NRP1* (Neuropilin-1) [32], and *PDIA6* (Protein Disulfide Isomerase Family A Member 6) [48] play crucial roles in cancer-related processes. The transcription factor MYC is overexpressed in many cancers and regulates the expression of multiple genes involved in the determination of cancer cell fate. Neuropilin-1 is a transmembrane protein also overexpressed in many tumors displaying growth-promoting functions and mediates tumor-initiating properties [49]. The disulfide isomerase PDIA6 has been shown to enable shedding of tumor-associated NKG2D (natural killer group 2, member D) ligands that promote resistance to cancer, thus contributing to tumor immune escape [50].

These numerous miR-127 and miR-376a target genes and their known functions in cancer-related processes confirm that therapeutic targeting of these miRNAs would influence multiple pathways that are crucial for the development and progression of cancers. Thus, silencing of these miRNAs and their demonstrated tumor suppressor function makes them suitable as therapeutic targets for novel osteosarcoma treatment strategies.

## 4. Materials and Methods

### 4.1. Tissue Samples and Cell Culture

All patients included in our study (*n* = 8) were diagnosed for osteosarcoma by open tumor biopsy between 2005 and 2012 and received equal neoadjuvant chemotherapy according to the standard recommendations within the EURAMOS-1 (European and American osteosarcoma study group) treatment study (Supplementary Table S1). Samples were used in an anonymous form under the approval of the ethical committee of the University of Heidelberg (S343-2015). Written consent was obtained from all patients included in this study.

Primary human osteoblasts (hOBs) were isolated from cancellous bone derived from patients without malignancies. After several washing steps with PBS (Thermo Fisher Scientific, Dreieich, Germany), bone fragments were cut into small pieces and digested in Dulbecco’s modified eagle medium (DMEM) high glucose (Biozym Scientific GmbH, Hessisch Oldendorf, Germany) supplemented with 1 mg/mL Collagenase Type II (Thermo Fisher Scientific) for 2 h at 37 °C under continuous rotation. After digestion, the fragments and already released cells were washed twice in PBS, transferred to a 0.1% gelatin-coated T75 cell culture flask, and cultivated in DMEM.

The osteosarcoma cell lines included in this study were 143B (#91112502) (Sigma-Aldrich, München, Germany), MNNG-HOS (#300289) (Cell Line Service GmbH, Eppelheim, Germany), CAL-72 (#ACC-439) (DSMZ, Braunschweig, Germany), MG63 (#CRL-1427) (American Tissue Culture Collection (ATCC), Rockville, Maryland, USA), U2OS (#300364) (Cell Line Service GmbH), and Saos-2 (#300331) (Cell Line Service GmbH). All cell lines were cultured in DMEM high glucose (Biozym) containing 4.5 g/L glucose and supplemented with 10% fetal calf serum (FCS) (Biochrom, Berlin, Germany), and 100 U/mL penicillin/streptomycin (Lonza GmbH, Köln, Germany).

### 4.2. Transfection of Osteosarcoma Cell Lines

For restoration of candidate miRNAs, osteosarcoma cell lines were transiently transfected with miR-127-3p (MIMAT0000446) or miR-376a-3p (MIMAT0000729) mimics (Life Technologies, Darmstadt, Germany) by electroporation. Control cells were electroporated without the addition of nucleic acids (WT = wildtype) or transfected with an miRNA mimic negative control (NC) (Life Technologies # 4464058) representing a random sequence molecule that has been extensively tested and validated to not produce identifiable effects on a known miRNA function. Transfection was carried out with the Neon Transfection Unit (Thermo Fisher Scientific). Cells were cultured until they reached ~80% confluence, trypsinized, and washed twice in phosphate buffered saline (PBS). For transfection, 10^6^ cells were resuspended in 100 µL R-buffer containing 1 µM microRNA mimic. After electroporation with two pulses at 1300 V for 15 ms, cells were plated in DMEM and cultured for 48 h before they were used for the different assays.

### 4.3. RNA Extraction, cDNA Synthesis, and RT-qPCR

For quantitative RT-PCR analyses of miRNA expression, total RNA was extracted from cultured cell lines, cultured hOBs, and cryo-conserved tumor samples. Frozen tissue was pulverized in a Micro-Dismembrator-S (Braun, Melsungen, Germany) and resuspended in TRI reagent included in the Direct-zol RNA isolation kit (Zymo Research, Freiburg, Germany). Cultured cells were washed in PBS and directly lyzed in TRI reagent. All further steps were performed according to the manufacturer’s protocol. RNA purities and concentrations were determined with a NanoDrop ND-100 spectrometer (PeqLAb, Erlangen, Germany).

Isolated RNA was reverse transcribed using the TaqMan MicroRNA Reverse Transkription kit from Applied Biosystems (Darmstadt, Germany) according to the manufacturer’s instructions. In brief, 10 ng of total RNA was subjected to cDNA synthesis using microRNA specific stem-loop primer. For RT-qPCR, 1.5 µL of cDNA was used in a total volume of 20 µL containing microRNA specific primers and TaqMan probes. Samples were heated to 95 °C for 10 min followed by 40 cycles of denaturation at 95 °C for 15 s and a combined annealing/extension step at 60 °C for 60 s. The reaction was carried out in the real-time thermal cycler LineGene 9600 (Biozym). Calculated microRNA expression levels were normalized on the basis of the RNU6B expression in corresponding samples. RNU6B is a small nuclear RNA frequently used as reference RNA for microRNA quantification.

### 4.4. Proliferation Assay

For the analysis of cell proliferation, 5 × 10^3^ cells were seeded in a 96-well plate 48 h after transfection, cultured in DMEM containing 10% FCS, and counted after 0, 24, 48, 72, and 96 h. For counting, cells were washed in PBS and trypsinized for 10 min at 37 °C. Trypsinization was stopped by the addition of DMEM/10% FCS and cells were transferred into a 96-well round bottom plate and counted using a MACSQuant flow cytometer (Miltenyi Biotec, Bergisch Gladbach, Germany). All analyses were done in triplicates.

### 4.5. Colony Formation Assay

Cells were trypsinized, counted, and resuspended in DMEM supplemented with 10% FCS, 100 U/mL penicillin/streptomycin, and 0.3% agarose (Biozym). The cell-agarose suspension (2.5 × 10^4^ cells in 300 µL) was layered on top of 300 µL 0.5% agarose in 24-well culture plates and incubated at 37 °C in a humidified incubator for 10 days. Colonies >500 µm^2^ were counted using ImageJ software. Experiments were done in triplicates with six different osteosarcoma cell lines.

### 4.6. Cell Cycle Analysis

Cells were washed in PBS, trypsinized, and counted. For each sample, 2 × 10^5^ cells were pelleted, resuspended in 200 µL PBS, and fixed by the addition of 1 mL of ice-cold 70% ethanol. Cells were incubated for 30 min at 4 °C, centrifuged, and resuspended in 1 mL of PBS supplemented with 0.1% Tween-20 (Carl Roth GmbH, Karlsruhe, Germany). Cells were centrifuged, resuspended in 200 µL PBS supplemented with 0.1% Triton-X 100 (Carl Roth GmbH), and 1 µL DNAse free RNase (Sigma-Aldrich, Taufkirchen, Germany) and incubated for 30 min at 37 °C. After the addition of propidiumiodide (Thermo Fisher Scientific) at a concentration of 2.5 µg/mL, cells were further incubated for 30 min at 37 °C before the DNA content of the cells was analyzed in a MacsQuant flow cytometer (Miltenyi Biotech).

### 4.7. Apoptosis Detection

Apoptotic cells were quantified using the caspase-3 substrate NucView 488 (Biotium, Fremont, USA) that becomes fluorescent upon enzymatic cleavage by active caspase-3. Transfected cells were seeded in a 24-well plate (7.5 × 10^4^ cells/well) and medium was replaced 24 h later to remove cells that died because of the electroporation procedure. Cells were then incubated for a further 72 h before culture supernatants containing dead cells and trypsinized vital cells were combined, washed in PBS, and centrifuged at 1000 rpm for 5 min. After staining for 30 min at 37 °C in the dark with NucView 488, cells were analysed in a MacsQuant flow cytometer (Miltenyi Biotech). As positive controls, cells were treated with 2 mM hydrogen peroxide (H_2_O_2_), a known inducer of apoptosis.

### 4.8. Chicken Chorioallantoic Membrane (CAM) Assay

Fertilized white Leghorn chicken eggs were delivered by a local ecological hatchery (Geflügelzucht Hockenberger, Eppingen, Germany). Upon delivery, eggs were cleaned with water and incubated in a motor breeder (Siepmann GmbH, Herdecke, Germany) at a humidity of 70%, a temperature of 37.8 °C, and permanent agitation. This time point was designated as embryonic development day 0 (EDD 0). At EDD4, eggs were prepared for transplantation by removal of 3 mL albumen and opening of the eggshell. This window of approximately a 1.5 cm diameter was not completely removed in order to close and reseal it with Leukosilk for further incubation. Transplantation of tumor cells was performed at EDD 9. The window was opened again and a sterile silicone ring (9 mm inner diameter) was placed onto the CAM. The CAM area within this ring was gently lacerated using a 30 gauge needle before 1 × 10^6^ cells resuspended in 20 µL medium were mixed with 20 µL Cultrex BME Type 3 matrix (AMS Biotechnology, Frankfurt, Germany) and inoculated onto the CAM. Seven days post-transplantation at EDD16, the xenograft tumors were resected after humane euthanasia of the chick embryo by injection of 50 µL of the pentobarbital Narcoren into the chicken vasculature. Embryos that died before EDD 16 were excluded from the study. The excised tumors were photographed and the volumes were estimated using the following formula: $volume=\left(4÷3\right)\times \pi \times r^{3}$ (r = ½ × √ diameter 1 × diameter 2). Tumor take rates were calculated as: Number of eggs with tumors * 100/number of eggs with vital embryos.

### 4.9. Data Analysis and Statistics

Statistical analyses were conducted using the SPSS software (IBM, Armonk, USA). Comparisons of two groups were performed by the Mann–Whitney U test. *p*-values below 0.05 were considered statistically significant and below 0.01 as highly significant. Reported *p*-values are two-sided.

## 5. Conclusions

In this study, we demonstrated the tumor suppressor functions of both miR-127-3p and miR-376a-3p in osteosarcoma cells, indicating that the observed silencing of these miRNAs is crucial for the maintenance of the neoplastic phenotype of these cells. Due to the numerous miR-127 and miR-376a target genes being closely related to important cancer-related processes, the targeting of these miRNAs might represent promising opportunities to improve current osteosarcoma therapy.

## Supplementary Materials

The following are available online at https://www.mdpi.com/2072-6694/11/12/2019/s1, Figure S1: Cell cycle analysis of osteosarcoma cells with restored miRNA expression. Table S1: Patient characteristics.
