# Supplementary material for: Tumor Suppressor Function of miR-127-3p and miR-376a-3p in Osteosarcoma Cells
Source: Cancers (Basel). 2019 Dec 14;11(12):2019. doi: 10.3390/cancers11122019 (PMC6966509; doi:10.3390/cancers11122019)
Supplement: Supplementary file 1 [file cancers-11-02019-s001.pdf]

Supplementary Materials: Tumor Suppressor Function of miR-127-3p and miR-376a-3p in Osteosarcoma Cells

Joerg Fellenberg, Burkhard Lehner, Heiner Saehr, Astrid Schenker and Pierre Kunz

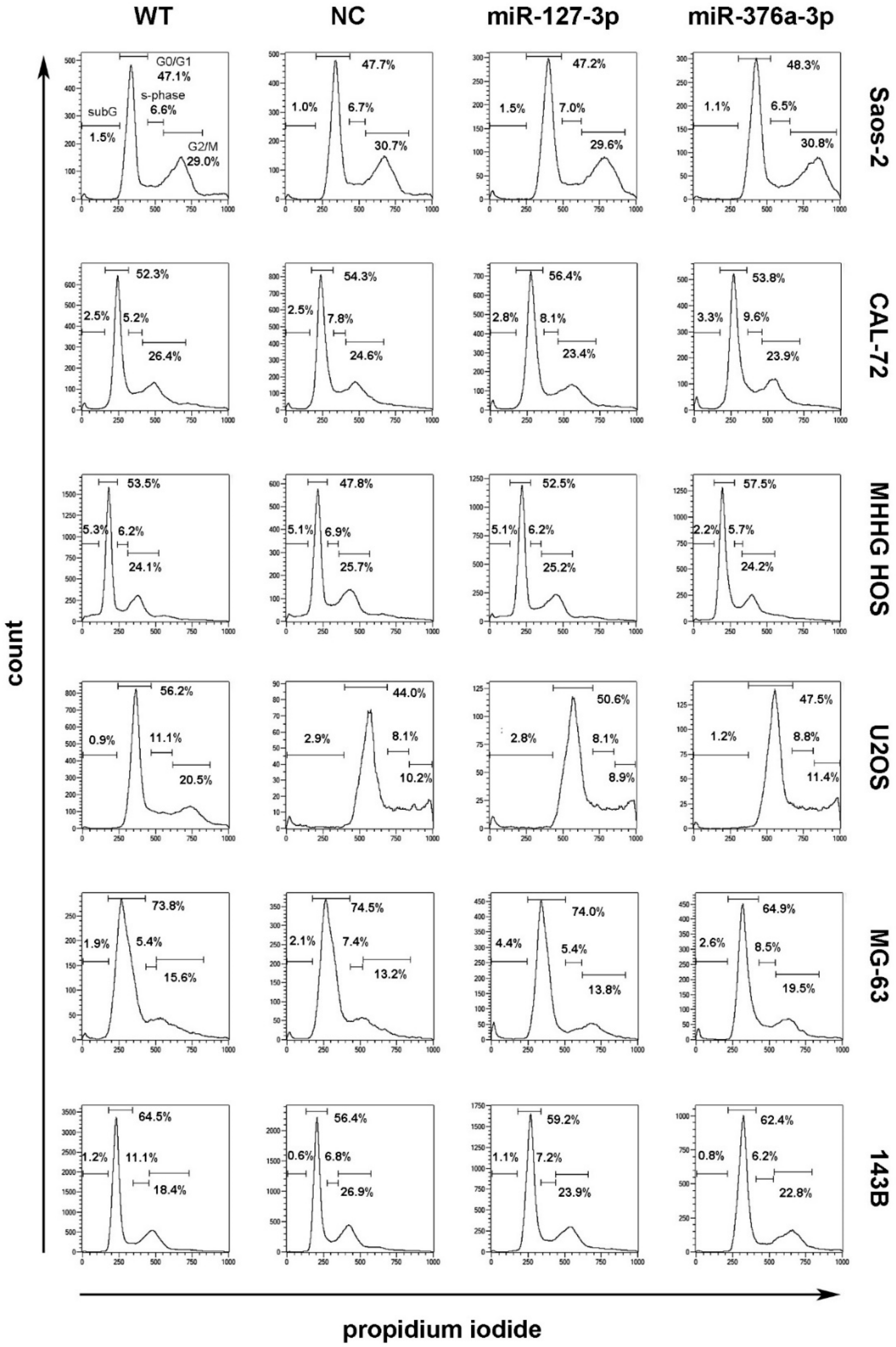

Figure S1. Cell cycle analysis of osteosarcoma cells with restored miRNA expression. The percentage of cells in the subG, G0/G1-, S- and G2/M-phase was determined 96 h after transfection

by flow cytometry. Representative graphs out of three independent experiments are shown for each cell line. (WT = wild type; NC = negative control miRNA).

Table S1. Patient characteristics.

ID	Gender	Age (years)	Max. Diameter (cm)	Localization	Subtype	Regression Grade	Metastases
OS1	f	14	9.5	femur	osteoblastic	4	-
OS2	f	39	13	femur	osteoblastic - fibroblastic	5	-
OS3	m	17	16	femur	osteoblastic - teleangiectactic	2	-
OS4	f	12	8.5	femur	chondroblastic	3	-
OS5	f	17	10	femur	osteoblastic	3	-
OS6	m	29	5	tibia	osteoblastic - chondroblastic	3	-
OS7	f	17	10	humerus	chondroblastic	4	-
OS8	m	21	14	tibia	osteoblastic	4	+

regression grade: according to Salzer Kuntschik (1 = good response; 6 = poor response).

© 2019 by the authors. Licensee MDPI, Basel, Switzerland. This article is an open access article distributed under the terms and conditions of the Creative Commons Attribution (CC BY) license (http://creativecommons.org/licenses/by/4.0/).
